# IDH Mutations in AML Patients; A higher Association with Intermediate Risk Cytogenetics

**DOI:** 10.31557/APJCP.2020.21.3.721

**Published:** 2020-03

**Authors:** Yasser H ElNahass, Ragia H Badawy, Fatma A ElRefaey, Hend A Nooh, Dalia Ibrahim, Heba A Nader, Hossam K Mahmoud, Wafaa H ElMetnawy

**Affiliations:** 1 *National Cancer Institute, Cairo University, Kasr Al Eini Street, Fom El Khalig, *; 2 *Genome Onco-Center, 52, Gameat Al Dowal Alarabya , Mohandeseen, *; 3 *Faculty of Medicine, Cairo University, Al-Saray Street, El Manial, Cairo, Egypt. *

**Keywords:** AML, normal karyotype, intermediate cytogenetic risk, IDH mutations, prognosis

## Abstract

**Objective:**

IDH mutations diversely affect the prognosis of cyogenetically normal acute myeloid leukemia (CN-AML) adult patients. The aim of this study is to assess the frequency of IDH mutations and to evaluate its role in AML prognosis.

**Methods:**

We have analyzed IDH1 and 2 mutations using High Resolution Melting curve analysis (HRM) in 70 denovo AML patients.

**Results:**

The median age of AML patients is 40 years (16-75). Incidence of IDH mutations is 10/70 (14.3%); 2 (2.9%) IDH1 mutant and 8 (11.4%) IDH2 mutant. Median PB blasts of mutant IDH patients was 67.5% (25-96) vs. 44% (0-98) for wild type (p=0.065). Eight/10 (80%) mutant IDH patients had B.M blasts ≥50% vs. 2/10 (20%) **<**50% (p**<**0.001) and were classified as intermediate risk cytogenetics (p=0.020) with wild FLT3-ITD (p=0.001). Ten/10 (100%) mutant IDH patients showed wild NPM1 (p=0.049). Median OS of mutant IDH in the intermediate risk cytogenetics was 1.8 years (0.7-3.1) vs. 3.1 years (1.1-5.5) for wild IDH (p=0.05).

**Conclusion:**

IDH mutation is mainly associated with intermediate risk AML and when integrated in this specific subgroup displays a lower survival and can be considered an additional integrated molecular risk marker for AML prognosis.

## Introduction

AML is a complex and dynamic disease whose prognosis depends upon a panel of cytogenetics and molecular abnormalities. The OS in AML is evaluated with a number of driver mutations as NPM1, CEBPA, FLT3-ITD, chromatin and RNA splicing genes, TP53 mutation, chromosomal aneuploidy all together with cytogenetic abnormalities and age. The specific mechanisms underlying leukemogenesis in AML are still poorly understood. Recently, there have been important advances in understanding AML pathogenesis (Döhner et al., 2015; Watts and Nimer 2018)

Comprehensive genomic analysis at diagnosis allows AML classification, risk stratification, prognosis, and permits for more selective therapeutic interventions (Thol et al., 2014; Metzeler et al., 2016; Papaemmanuil et al., 2016). In addition molecular profiling has a particularly important role in re-categorizing patients with CN-AML representing half of the newly diagnosed AML patients (Niparuck et al., 2019). CN-AML with mutated NPM1 or a mutated CEBPA, in the absence of FLT3-ITD, has been considered as a low risk AML (Niparuck et al., 2019). The favorable prognostic impact of CEBPA mutations has been further refined to biallelic mutations only (Li et al., 2015).

Isocitrate dehydrogenases (IDH1 and 2) are enzymes involved in multiple metabolic and epigenetic cellular processes (Willander et al., 2014). Mutations in IDH1 or IDH2 are detected in approximately 20% of AML patients inducing amino acid changes in conserved residues resulting in neomorphic enzymatic function and production of an oncometabolite, 2-hydroxyglutarate, leading to DNA hypermethylation, aberrant gene expression, cell proliferation and abnormal differentiation (Dang et al., 2016; Montalban-Bravo and DiNardo 2018) . Somatic mutations in IDH1 and IDH2 occur as early clonal events in AML evolution (Chou et al., 2012; Corces-Zimmerman et al., 2014; Shlush et al., 2014; Molenaar et al., 2015; Papaemmanuil et al., 2016).

Prognosis of AML patients can be diversely affected by IDH mutations and other co-occurring mutations. Recently, specific targeted therapies against mutant IDH have offered novel lines of therapy for AML patients (Lu et al., 2012; Wang et al., 2013; Kernytsky et al., 2015; Papaemmanuil et al., 2016; Xu et al., 2017; Olarte et al., 2019). 

This study was undertaken to assess the frequency of IDH1 and IDH2 mutations in AML patients and evaluate its role in disease prognosis.

## Materials and Methods

*Patients*

Seventy denovo AML patients were presented to Hematology Department at National Cancer Institute (NCI). Patients were diagnosed as AML based on morphology, cytochemistry, immunophenotyping, routine cytogenetic analysis, and routine molecular detection of NPM1, FLT3-ITD, t(8;21), PML/RARA and inv16q by PCR (Polymerase Chain Reaction). The patients were classified into low, intermediate and high risk groups according to ELN 2017 classification (Dohner et al., 2017). Diagnosis was established according to WHO criteria 2016 (Arber et al., 2017). Patients who are 16 years or older were treated with the adult AML regimen protocol at our Institute, and were included in our study. APL and patients with history of AML treatment were excluded. “Median follow up was 3.7 years (1.02-6.5). All patients gave written informed consent and the study was approved by the Institutional Review Board according to Helsenki.

*Methods*

*IDH Mutations By High-Resolution Melting (HRM) Analysis*

Twenty nanogram of DNA were amplified in a final volume of 10 uL containing 1X High Resolution Melting PCR Master Mix (Type it, Qiagen) with a saturating fluorescent DNA-binding dye, 0.2mM of each primer and 2.5mM MgCl_2_. Primer sequences were (forward IDH1: 5'-ccatttgtctgaaaaactttgcttct-3', reverse IDH1: 5'-tcacattattgccaacatgactt-3', forward IDH2: 5'-tctggttgaaagatggcggc-3' and reversed IDH2: 5'-caagaggatggctaggcgag-3'). One positive control and one non- template control were included in each experiment. All samples were tested in duplicate. Cyclic parameters were as follows: initial denaturation at 95ºC for 10 min; 45 cycles of 95ºC for 10 s, 58ºC for 10 s and 72ºC for 20 s. Final melting program was denaturation at 95ºC for 1min, renaturation at 45ºC for 1 min and melting from 60ºC to 95ºC with a ramp of 0.02ºC/sec and 25 fluorescence acquisitions/ºC (Ibáñez et al., 2012). All reactions were performed in duplicate. Wild-type and mutated samples were defined as positive and negative controls in the software. All HRM results were analyzed as fluorescence versus temperature graphs by Eco Illumina software (San Diego, CA) with normalized, temperature-shifted melting curves displayed as difference plot.

*Statistical Methods*

Statistical analysis was done using IBM SPSS® Statistics version 22 (IBM^®^ Corp., Armonk, NY, USA). Numerical data were expressed as mean and standard deviation or median and range as appropriate. Qualitative data were expressed as frequency and percentage. Pearson’s Chi-square test or Fisher’s exact test was used to qualitative variables. Mann-Whitney test was used for the non normally distributed quantitative data (non parametric t-test). Survival of AML patients was done using Kaplan-Meier method and comparison between two survival curves was performed using the log-rank test. All tests were two-tailed. A p-value < 0.05 was considered significant.

## Results

Seventy newly diagnosed AML patients, 33 males (47.1%) and 37 females (52.9%) with median age of 40 years (16-75) were included. Median Hb was 7.1 gm/dL (5.1-11.5), median TLC was 30.5 x10^9^/L (0.24- 409), median Platelet Count was 42 x10^9^/L (3.0-537), median P.B blasts were 58 (0-98) and median B.M blasts were 60 (37 - 90).

Fifty three /70 (75.7%) patients were CN- AML and 17 (24.3%) patients showed an abnormal karyotype. (Table 1).

*Molecular Mutations*

IDH mutations occurred in 10/70 (14.3%) patients. IDH1&2 were mutually exclusive, IDH1 mutation was found in 2/70 (2.9%) patients, while IDH2 mutation was found in 8/70 (11.4%) patients. 

FLT3-ITD mutation was positive in 12/70 (17.1%) patients. NPM1 mutation was positive in 19/40 (47.5%) patients and both mutations co-occurred in 4/40 (10%) patients.

According to ELN 2017 classification, which categorized the patients into 3 risk groups according to molecular and cyogenetic profile. In our study according to this classification, normal karyotype AML constitutes 53/70 (75.7%) of the whole AML group. However, they were not all classified as intermediate risk group, as further genetic refinement of the patients genetic profile like NPM1, FLT3-ITD and C-KIT mutations changed patients classification from intermediate risk 33/70 (47.1%) to either low risk 24/70 (34.4%) (including 9 patients with CBF leukemia and 15 patients with NPM1 mutant) or high risk groups 13/70 (18.6%) patients (including 12 patients with FLT3-ITD mutant and one patient with -7).

Relation Between IDH Mutations And Lab. Parameters

Median PB blasts % of mutant IDH which was 67.5 % (25-96) vs. 44 % (0 - 98) for wild type IDH (p=0.065).

Eight/10 (80%) mutant IDH patients had B.M blasts ≥ 50% vs. only 2/10 (20%) wild type patients (p >0.001). No statistical relation could be found between IDH gene mutations and different immunophenotypic aberrant markers expression including *CD2*, *CD7*, *TDT* or with *CD123* expression.

Nine/10 (90%) IDH mutant patients were CN-AML, while one IDH mutant patient had trisomy 8. FLT3-ITD showed mutual exclusivity with IDH gene mutation; 8/10 (80%) mutant IDH was wild for FLT3-ITD (p=0.001). NPM1 mutation showed a statistical association with wild type IDH as all NPM1 mutant patients 19/19 (100%) had wild type IDH (p=0.049) (Table 2).

All mutant IDH patients were negative for CBF translocations (t8;21/ inv16q). We observed a strong relation between IDH mutations and cytogenetic risk group as 8/10 (80%) mutant IDH patients belonged to the intermediate risk group (p= 0.020) (Table 3).

Response to treatment analysis involved 42/70 (60%) patients only as 22/70 (31.4%) patients had early deaths (before completing the induction treatment and died before day 28). On the 28^th^ day of induction chemotherapy, 34/42 (81%) patients achieved morphological complete remission.

The median follow up period was 3.7 years (1.02-6.5) after exclusion of early deaths. At the end of the study 52/70 (74% ) patients died. Median OS was 6.4 years.

Median survival of AML patients with P.B blasts <50% was 3.3 years (1.4-5.5) vs. 1.8 years (0.8-2.8) years with P.B blasts ≥ 50% (p=0.03).

There was a trend significant relation between IDH mutation and survival, where OS of IDH mutant patients were 1.8 (0.69-3.15) years vs. 3.1 (1.1-5.1) years for the wild IDH (p=0.089). 

OS of IDH mutant patients in the intermediate risk group was inferior to wild type patients; median OS was 1.8 years (0.69-3.15) vs. 3.2 years (1.3-5.1) respectively (p=0.05).

**Table 1:** Cytogenetic Abnormalities in AML Patients

	n (%)
Normal Karyotype	53 (75.7%)
Abnormal Karyotype:	17 (24.3%)
t (8;21)	8 (11.4%)
inv16q	3 (4.3%)
45,XY,-7	1 (1.4%)
48,XX, +8, +19	1 (1.4%)
47,XX,+4	1 (1.4%)
47,XX,+8	1 (1.4%)
47,XX, +19	1 (1.4%)
46,XX,+14,-10	1 (1.4%)

**Table 2:** Association between IDH Mutations and FLT3-ITD & NPM1

	Wild IDH (n= 60)	Mutant IDH (n=10)	p value
FLT3-ITD			0.001
Wild (n =58)	50 (86.2%)	8 (13.8%)	
Mutant (n=12)	10 (83.3%)	2 (16.7%)	
NPM1			0.049
Wild (n=21)	16 (76.2%)	5 (23.8%)	
Mutant (n=19)	19 (100%)	0 (0.0%)	

**Table 3:** Association between IDH Mutation and Risk Stratification of AML Patients

	Low Risk (n = 24)	Intermediate Risk (n = 33)	High Risk (n = 13)
Wild IDH (n= 60)	24 (40%)	25 (41.6%)	11 (18.4%)
Mutant IDH (n= 10)	0 (0%)	8 (80%)	2 (20%)
p-value	<0.01	0.02	0.5

## Discussion

AML risk stratification remains challenging for about 50% of patients with CN-AML which is associated with either favorable or intermediate risk (Papaemmanuil et al., 2016). This group of AML patients is challenging to stratify, and, accordingly, further molecular mutations are required. AML is a disease with a heterogenic nature. Different molecular and cytogenetic signatures change the disease nature, prognosis and response to treatment. As for all the studied AML patients, routine cytogenetic and molecular analysis were done. However, patients with CBF leukemia who were found to be C-KIT positive are reclassified as intermediate risk (O’Donnell et al., 2012).

In this study, we have analyzed the mutations of *IDH1* and* 2* genes to evaluate their prognostic values in the newly diagnosed AML patients. Overall incidence of *IDH* mutations was 14.3%, 2/70 (2.9%)* IDH1 *mutant and 8/70 (11.4 %) *IDH2* mutant. This incidence is in agreement with other reports (Papaemmanuil et al., 2016; Montalban-Bravo and DiNardo 2018). However, in another study, *IDH1* and *2* mutations were detected in 5.5% and 4%, respectively (Raveendran et al., 2015). Some reports found the frequency of *IDH1* mutations in AML patients from various countries 2-14% (Chotirat et al., 2012; Ahmad et al., 2014). In the present study, we have found that *IDH1* and *IDH2* mutations were mutually exclusive as previously reported (Papaemmanuil et al., 2016). We have correlated *IDH* mutations with patient characteristics, different laboratory findings and AML prognostic factors. There was a female predominance for *IDH* mutations (3M/7F), but the difference was not significant and this result was in agreement with another report (Raveendran et al., 2015). We have not found a significant relation between IDH mutations and Hb concentration, TLC and platelet count (p=0.924 , 0.611 and 0.0935 respectively) which was in agreement with another report (Patel et al., 2011).

IDH mutations were associated with older age. Median age of mutant IDH patients were 46.5 yrs (26-75) vs. 37 yrs (16-70) of the wild IDH (p=0.29). Median PB blasts % was 67.5% (25-96) with IDH mutant patients vs. 44% (0-98) with wild IDH (p=0.065) which were in agreement with previous reports (DiNardo et al., 2015; DiNardo t al., 2016).

Like some other researchers, we have found that IDH mutations were more frequently observed in patients with intermediate risk cytogenetics (8/10, 80%) (p=0.020) and are particularly frequent in CN-AML (Cancer Genome Atlas Research Network et al., 2013; Aref et al., 2015). In this study, 80% of IDH mutant AML belonged to the intermediate risk group which was significant to us as only 20% mutant IDH belonged to high risk category. However, when looking at the intermediate risk category 8/33 (24%) of patients were mutant for IDH vs. 0/24 (0%) in the low risk category and 2/13 (15%) only in the high risk cytogenetic group. This shows that there is a higher association between IDH mutation and the intermediate risk cytogenetics AML. However, a larger sample of our AML patients is recruited to prove such association”. FLT3-ITD was negative in 8/10 (80%) of IDH mutant patients with (p=0.001). This result was in agreement with others who found that IDH mutations were not associated with FLT3-ITD mutations (Marcucci et al., 2010; Virijevic et al., 2016). However, these results contradict many other reports that found FLT3-ITD mutated AML associated with IDH mutations (DiNardo et al., 2016; Papaemmanuil et al., 2016; Boddu et al., 2017), which could be attributed to ethnic variations and other genetic markers interactions.

All patients with mutant IDH genes had wild NPM1 (p=0.049). However, we could not draw a conclusion as only 40/70 patients had results for NPM1 molecular status. This striking observation was in discordance with major leading reports about the genomic landscape of AML (DiNardo et al., 2016; Papaemmanuil et al., 2016) who found that the frequency of co-occurring NPM1 mutation seems higher in the presence of IDH1/2 mutations (65% vs. 48%). If this could be translated to a different disease, biology in our AML patients is still a point of discussion and needs a higher sample size for evaluation. In another major study about the spectrum and prognostic relevance of driver gene mutations in AML, IDH2 mutations were not found to be associated with NPM1 mutations and only IDH1 mutations was weakly pair wise associated with FLT3-ITD (Metzeler et al., 2016). These results were also in agreement with reports stating that IDH2 mutated patients displayed infrequent NPM1 mutations and lower WBC count (DiNardo et al., 2016). Like others, we have found that IDH mutations were mutually exclusive with CBF AML (Raveendran et al., 2015). 

Response rate in our study was not impacted by the IDH mutational status because the treatment strategies received were heterogeneous and were dependent on several factors such as patient age, performance status, co-morbidities, and therapies received prior to referral to our institution. According to Döhner et al., 2010, occurrence of CR is observed on 28th day of starting the chemotherapy protocol. Relapse was defined by ≥ 5% BM blasts, reappearance of circulating leukemic blasts, or development of extra medullary leukemia. Therefore, the exclusion of early deaths due to sepsis, hemorrhage or chemotherapy complication was essentially done to avoid false misleading results about the effect of IDH mutation on disease burden and patient survival, which if included, a lower overall survival would be reflected to IDH mutation. Patients with P.B blasts ≥50% displayed a lower OS than patients showing <50% (6.4 years vs. 1.8 years) (p=0.03) and this was attributed to associated IDH mutations. In addition, there was a trend significant relation between IDH mutation & OS where wild type IDH had a cumulative survival at 6 years of 55% vs. 28.6% for mutant IDH (p=0.089). These results were in agreement with another report (Xu et al., 2017).

Our results are in agreement with major leading studies regarding IDHR132 & IDH2R140 regarding OS (Marcucci et al., 2010; Feng et al., 2012; Papaemmanuil et al., 2016; Montalban-Bravo and DiNardo 2018). In this work, when we integrated IDH results with the intermediate risk cytogenetics, we remarkably found that the OS of the intermediate risk AML group was inferior for mutant IDH patients in comparison with wild IDH patients (median OS 1.8 years vs. 6.4 years, respectively p > 0.05). 

In conclusion, IDH mutations detection should be integrated into AML prognostic panel in the new era of therapeutic modalities. Incidence of IDH mutations is mainly associated with CN-AML. When integrated into this specific subgroup category, it displays a lower survival, and, thus it can be considered an additional integrated molecular risk marker of AML prognosis within the normal/ intermediate cytogenetic group. In countries of limited resources, HRM is an alternative and more rapid and cost effective method of detection of gene mutation than the sequencing methods. 
